# Efficient production and transmission of CRISPR/Cas9-mediated mutant alleles at the IG-DMR via generation of mosaic mice using a modified 2CC method

**DOI:** 10.1038/s41598-019-56676-5

**Published:** 2019-12-27

**Authors:** Satoshi Hara, Miho Terao, Akari Muramatsu, Shuji Takada

**Affiliations:** 1 Department of Systems BioMedicine, National Research Institute for Child Health and Development, Tokyo, 157-8535 Japan; 2 Division of Molecular Genetics & Epigenetics, Department of Biomolecular Sciences, Faculty of Medicine, Saga University, Saga, 849-8501 Japan

**Keywords:** CRISPR-Cas9 genome editing, Imprinting

## Abstract

Generation of mutant imprinting control region (ICR) mice using genome editing is an important approach for elucidating ICR functions. IG-DMR is an ICR in the *Dlk1*-*Dio3* imprinted domain that contains functional regions—in both parental alleles—that are essential for embryonic development. One drawback of this approach is that embryonic lethality can occur from aberrant expression of the imprinted genes if IG-DMR gets mutated in either the paternal or maternal allele. To overcome this problem, we generated mosaic mice that contained cells with modified IG-DMR alleles and wild-type cells using the 2CC method that allowed for microinjection of the CRISPR/Cas9 constructs into a blastomere of 2-cell embryos. This method improved the birth rate of the founder pups relative to that obtained using the standard protocol. We also successfully produced mosaic mice in which the tandem repeat array sequence in the IG-DMR had been replaced by homology directed repair. Additionally, paternal transmission of the replaced allele caused aberrant expression of the imprinted genes due to hypomethylation of the IG-DMR, indicating that the replaced allele recapitulated our deletion model. Our results indicate that this method is useful for the generation of mutant mice in which a genomic locus essential for normal development has been genetically edited.

## Introduction

It is generally accepted that the RNA-guided clustered regularly interspaced short palindrome repeat-associated Cas9 nuclease (CRISPR/Cas9) system is the most rapid, efficient, and cost-effective genome editing tool. The CRISPR/Cas9 system consists of a single-guide RNA (sgRNA) for the recognition of a target genomic locus, and Cas9 for binding to the sgRNA and digestion of DNA. A double-stranded break (DSB) generated by sgRNA/Cas9 at a target site induces non-homologous end-joining (NHEJ) repair, and errors in this process cause insertion or deletion (indel) mutations, or a large deletion. Alternatively, homology-directed repair (HDR) induced by knock-in when a donor DNA template containing an exogenous DNA sequence and homology arms is co-introduced along with sgRNA/Cas9^1–3^.

In general, genetically modified mice can be generated with the CRISPR/Cas9 system by microinjection of *in vitro*-transcribed sgRNA and the Cas9 mRNA/protein into the cytoplasm of fertilized eggs^4,5^. The founder mice produced by this method are not suitable for phenotype analyses because they include heterozygous, homozygous, and mosaic mutants in which genome editing occurred after DNA replication during the S-phase in fertilized eggs. Therefore, it is difficult to determine whether mutants are mosaic by standard genotyping protocols that use PCR from small biopsy samples such as tail tips^6^. To overcome this issue, it is essential to generate homozygous mice by crossing F_0_ or F_1_ mutants for precise phenotype analyses, providing that the mutants are fertile.

A subset of genes specifically expressed by the parent-of-origin are said to be imprinted^7^. This epigenetic phenomenon is known as genomic imprinting and is associated with embryonic development and placenta formation, as well as with several disorders. In mammalian genomes, many imprinted genes form clusters and are regulated by an imprinting control region (ICR), which is a key regulatory sequence for an entire cluster. ICRs possess differential functions in paternal and maternal alleles due to epigenetic modifications of parental origin^8^. To understand the molecular mechanisms underlying ICR regulation of imprinted genes, genetic modification of ICRs is an important approach. However, genetic modification of an ICR on a functional allele may result in aberrant expression of imprinted genes in the founder mice, but might have no effect on the other allele. In cases of genetic modification of a functional allele, this can lead to founder lethality (e.g., the *Dlk1*-*Gtl2* intergenic differentially methylated region (IG-DMR), which is an ICR for the *Dlk1*-*Dio3* imprinted cluster, or the *Nespas*-*Gnasxl* DMR, which is an ICR for the *Gnas* imprinted cluster)^9,10^. In particular, it is known that the deletion of a 4.1-kb region within the IG-DMR leads to perinatal lethality when it is transmitted maternally. We previously reported that a 216-bp tandem repeat array sequence within the 4.1-kb region of the IG-DMR (IG-DMR-Rep) is essential for the imprinting status of IG-DMR as a paternal allele, suggesting that there is a risk for embryonic lethality when either the methylated-paternal or the unmethylated-maternal allele of IG-DMR is mutated through genome editing^11^. In addition, epigenetic modifications in ICRs are erased and reprogrammed in a sex-dependent manner; therefore, F_0_ mice with mutated ICRs may not always transfer the mutant allele to the F_1_ generations, due to lethality or sterility if the ICR is functional when it is derived from the father and the pups are male, and *vice versa*^12^. Similar situations also occur during genome editing when the presence of heterozygous mutations can cause lethality or infertility in the founder mice, such as that observed upon editing dosage-sensitive (e.g. *Sox9*) and sex-linked genes (e.g., *Sry* and *Zfy1/2*)^13–15^.

Recently, Wang *et al*. generated mosaic mice carrying both *Tet3*-deficient and wild-type cells by microinjecting Cas9 mRNA into fertilized eggs, followed by microinjection of sgRNA into a blastomere at the 2-cell stage (termed 2-cell embryo-CRISPR-Cas9 injection [2CC method]) to avoid embryonic lethality from *Tet3* deficiency^16^. Although this is a useful method for introducing mutations in genes—essential for development—through NHEJ, it is unclear if cells containing the genetically modified alleles can contribute to the germ cell pool in the resultant mosaic mice, and if so, what percentage of offspring contains the mutated allele. In addition, Gu *et al*. have reported that HDR-mediated knock-in mice can be generated by injecting sgRNA/Cas9 and a plasmid containing the donor sequence into 2-cell stage embryos^17^. This implies that heritable founder mice carrying an HDR-mediated knock-in in the ICR can be efficiently generated by microinjection of sgRNA/Cas9 along with a donor template DNA into a blastomere in the 2-cell stage embryo.

In this study, we aimed to determine whether the low birth rates in founder mice in which the IG-DMR was targeted can be improved by generating artificial mosaic mice using the 2CC method with minor modifications (modified 2CC). Specifically, this modified 2CC incorporates microinjection of sgRNAs and the Cas9 protein only at the 2-cell stage. Furthermore, we examined the efficiency of deletion allele transmission to the next generation, where deletion of a paternal, but not maternal, allele causes perinatal lethality. Additionally, we demonstrated that the modified 2CC method can be applied to the creation of HDR-mediated knock-in mice in which IG-DMR-Rep has been replaced with an endogenous CpG-free sequence. Analysis of embryos containing a paternally transmitted replaced allele revealed that paternal IG-DMR was hypomethylated, the expression of imprinted genes was aberrant, and these changes were embryonic-lethal. Observed phenotypes were the same as in embryos possessing a paternally transmitted deletion of the IG-DMR-Rep, suggesting that those phenotypes result from a loss of methylated IG-DMR-Rep, and not from conformational changes caused by IG-DMR-Rep deletion.

## Results

### The modified 2CC method improved the birth rate of founder pups carrying a deletion of the IG-DMR

**Figure 1:**
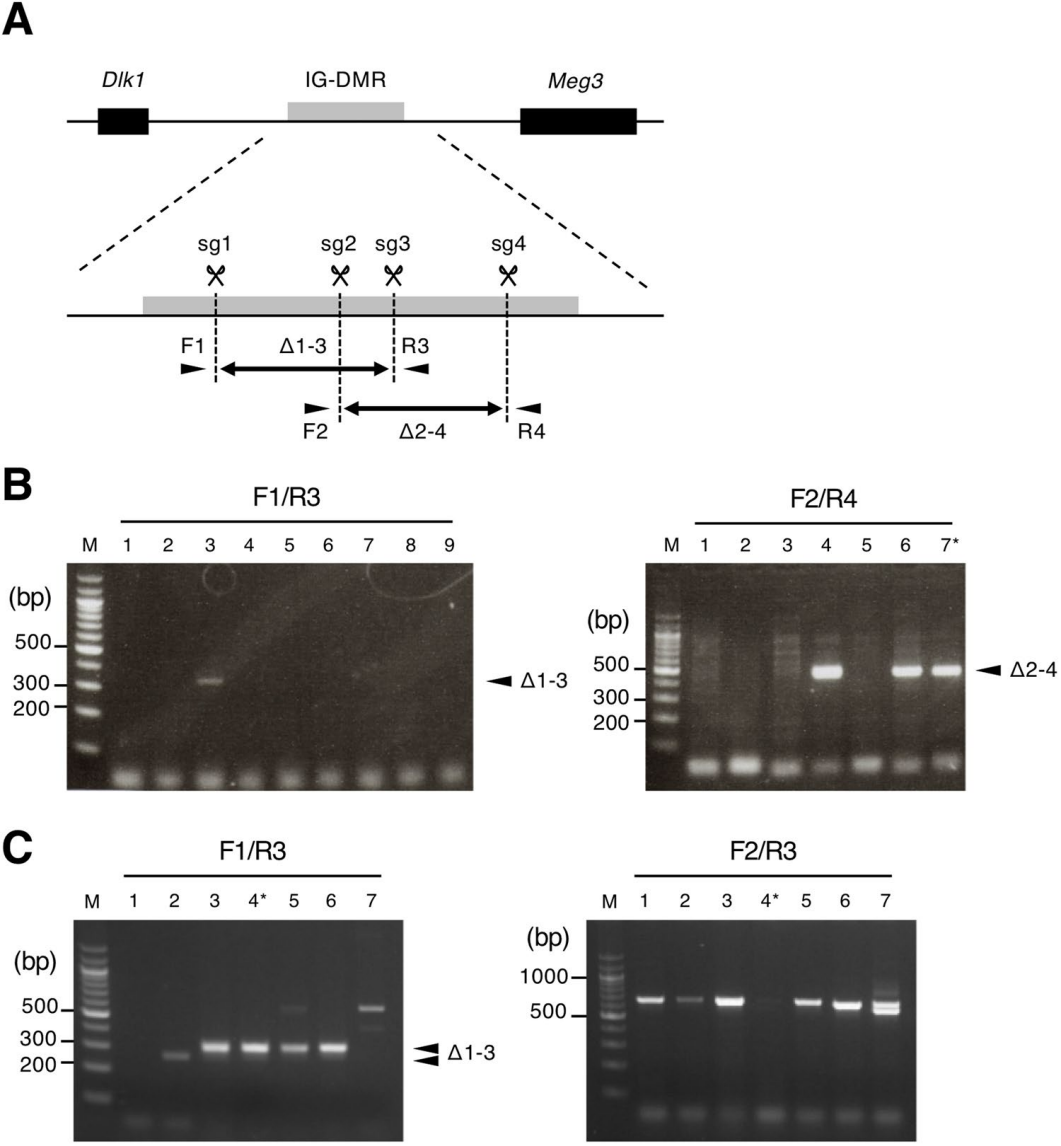
Introduction of a deletion at the IG-DMR locus by microinjecting sgRNAs/Cas9 into 1-cell embryos. (**A**) Schematic images of mouse *Dlk1*-*Gtl2* locus. Genomic DNA and genes are indicated with a black line and boxes, respectively. IG-DMR is indicated with a gray box. Scissors and double-headed arrows indicate target sequences for sgRNA and deleted regions, respectively. Genotyping primers are shown by arrowheads. (**B**) Genotyping analysis of F_0_ pups. Agarose gel electrophoresis images are shown. Primer pairs used are shown at the top of each photo. Numbers indicate mice IDs. PCR products amplified from Δ1–3 and Δ2–4 alleles are shown with arrowheads. An asterisk indicates an F_0_ pup that died within 10 days after birth. M: 100 bp ladder marker. (**C**) Genotyping analysis of F_0_ embryos injected with sg1/sg3/Cas9 at 16 days after transfer. Representative agarose gel electrophoresis images are shown. Numbers indicate mice IDs. Arrowheads indicate PCR products amplified from Δ1–3. Asterisk indicates the embryos considered homozygous deletions. M: 100 bp ladder marker. Full agarose gel images of Fig. 1B,C are shown in Supplementary Fig. S4.

As a model of genome editing at ICRs, we chose IG-DMR in the *Dlk1*-*Dio3* domain. Four sgRNAs (sg1-4) were designed to target the IG-DMR locus (Fig. 1A). To produce mice carrying two deletion patterns at the IG-DMR, we injected Cas9 and sg1/sg3 or sg2/sg4 into 78 and 112 zygotes, respectively. Fifty-six embryos at the 2-cell stage were injected with sg1/sg3/Cas9, and 88 with sg2/sg4/Cas9, which were subsequently transferred into pseudo-pregnant female mice. Nine (16%) and 6 (7%) live pups were obtained from sg1/sg3/Cas9 and sg2/sg4/Cas9 zygotes, respectively, by Caesarian operation on the recipient females at 19 days post transfer. Genotyping analysis was used to detect PCR products that were deletion mutants; this showed that 1 out of 9 sg1/sg3/Cas9 pups and 3 out of 7 sg2/sg4/Cas9 pups carried the deletion allele for IG-DMR (Fig. 1B). However, 1 sg2/sg4/Cas9 pup carrying the deletion allele exhibited severe growth retardation and died within 20 days after birth. Finally, only 1 out of 9 sg1/sg3/Cas9 pups and 2 out of 6 sg2/sg4/Cas9 pups carrying deletion alleles for IG-DMR grew to adulthood (Table 1). To test whether the low efficiency of the deletion in pups injected with sgRNAs/Cas9 was the result of embryonic lethality, we microinjected sg1/sg3/Cas9 into fertilized eggs and analyzed the genotype at late-stage gestation. Nineteen embryos were obtained at 16 days after embryo transfer. PCR genotyping analysis using the F1/R3 primer pair showed that 16 embryos carried the deletion while primer pair F2/R3, which indicates the existence of a partial sequence between the sg1 and sg3 regions, showed no amplification in 3 out of 19 embryos, suggesting that these were homozygous carriers for a deletion between sg1 and sg3 (Fig. 1C). As the efficiency of the deletion was higher in embryos than in newborns, this implies that most embryos carrying the deletion allele die between late gestation and delivery, possibly due to the loss of imprinting brought about by deletion of a functional sequence within IG-DMR.

**Table 1:** Generation of mice with a deletion at IG-DMR using the modified 2CC method.

sgRNA/Cas9 combination	Injection protocol	No. of Injected embryos	No. of Transferred embryos (litter no.)	No. of surviving F_0_ pups (survival rate)^a^	No. of pups with deletion^b^	Total No. of F_0_ pups that grew to adulthood
sg1/sg3/Cas9 mRNA	standard	78	56 (3)	9 (16%)	1 (11%)	1
sg1/sg3/Cas9 protein	2CC	60	48 (2)	24 (50%)	5 (18%)	4
sg2/sg4/Cas9 mRNA	standard	112	88 (4)	7 (8%)	3 (33%)	2
sg2/sg4/Cas9 protein	2CC	61	48 (2)	19 (24%)	5 (40%)	5

^a^Survival rate indicates percentage of living pups to transferred 2-cell embryos.

^b^Parentheses indicates percentage of pups with deletion allele to total no. of surviving pups.

**Figure 2:**
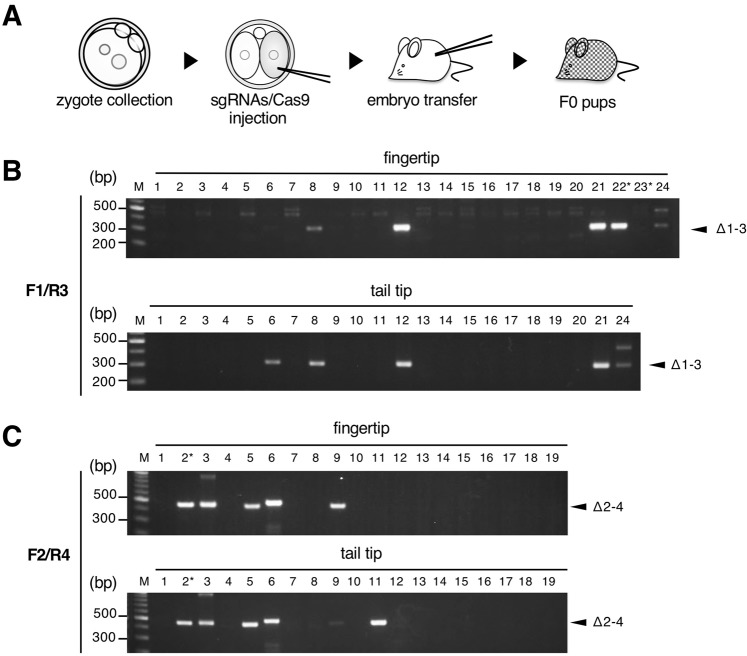
Deletion at IG-DMR locus using the modified 2CC method. (**A**) Schematic representation of experimental protocol. sgRNA/Cas9 complex was injected into the cytoplasm of single blastomeres. After embryo transfer, resulting mosaic F_0_ pups were subjected to genotyping analysis. (**B**) Genotyping analysis of F_0_ pups injected with sg1/sg3/Cas9. Agarose gel electrophoresis images of PCR products amplified from newborn fingertips and tail tips at P5 are shown at top and bottom, respectively. The PCR primer pair used is indicated at top left. Numbers indicate mouse ID. Asterisks show mice that died within 10 days after birth. Arrowhead indicates the target PCR product. M: 100 bp ladder marker. (**C**) Genotyping analysis of F_0_ pups injected with sg2/sg4/Cas9. Agarose gel electrophoresis images of PCR products amplified from newborn fingertips and tail tips at P5 are shown at top and bottom, respectively. The PCR primer pair used is indicated at top left. Numbers indicate mouse ID. Asterisks show mice that died within 10 days after birth. Arrowhead indicates the target PCR product. M: 100 bp ladder marker. Full agarose gel images for Fig. 2B,C are shown in Supplementary Fig. S4.

To improve the number and survival rate of F_0_ pups with IG-DMR deletion, we hypothesized that mosaicism between cells with deletion alleles and wild-type cells could circumvent the lethality caused by deletion of IG-DMR. To test this, we injected sgRNA pairs (sg1/sg3 or sg2/sg4) and Cas9 with an added nuclear localization signal into the cytoplasm of a blastomere of the 2-cell stage embryos (Fig. 2A). Sixty embryos injected with sg1/sg3/Cas9 and 61 embryos injected with sg2/sg4/Cas9 were transferred to pseudo-pregnant mice. Nineteen days after transfer, we obtained 24 (50%) living pups from sg1/sg3/Cas9 embryos and 19 (40%) pups from sg2/sg4/Cas9 embryos. Genotyping analysis of fingertip DNA from the resulting pups showed that 5 out of 24, and 5 out of 19 pups were mutants carrying a deletion allele between sg1 and sg3, (Δ1-3) and between sg2 and sg4 (Δ2–4), respectively (Figs. 2B and S1). The survival rate of sg1/sg3 embryos (the number of surviving pups relative to total transferred embryos) was significantly higher when using the modified 2 CC as compared to the standard protocol (modified 2 CC vs standard; 50% vs 16%, *P* < 0.01, Fisher’s exact test). A similar trend was also observed in sg2/sg4 embryos (modified 2CC vs standard; 50% vs 16%, *P* < 0.01, Fisher’s exact test). Importantly, the percentage of mice with deletions in the surviving pups was similar upon using either modified 2CC or standard methods, possibly because the NHEJ-mediated deletion occurred with similar efficiency.

Next, we re-genotyped the mutants using the same method, but with DNA prepared from tail tips, to roughly analyze the mosaicism. When re-genotyping Δ1–3 mutants using this method, the PCR products indicated the existence of Δ1–3 alleles in 4 out of 5 pups. In addition, a Δ1–3 allele was detected in the sample from the tail tip of mouse #6, which showed no detectable PCR product in the sample prepared from fingertip, suggesting that the contribution of cells carrying the deletion allele was slightly lower in #6 as compared to the others, or that the ratios of mutant to wild-type cells are more biased in tissues from #6 than those from the others (Fig. 2B). Similarly, Δ2–4 alleles were detected in both or either fingertip and tail tip samples from four (#2, #3, #5 and #6) or two pups (#9 and #11), respectively. From pups #9 and #11, the Δ2–4 allele was detected only in fingertip or only in tail DNA, respectively (Fig. 2C). Finally, 18% (4 out of 22) of the sg1/sg3/Cas9 pups and 28% (5 out of 18) of the sg2/sg4/Cas9 pups carrying deletion alleles grew to 4-weeks-old, indistinguishable from wild-type (Table 1), suggesting that these efficiencies were comparable to the rates obtained by microinjection of sg1/sg3/Cas9 and sg2/sg4/Cas9 into zygotes (Table 1, Fig. 2B,C). These results showed that generation and survival rate of pups with deleted IG-DMR were improved via mosaicism induced using the modified 2CC method.

To assess the germline contributions of cells with deletion alleles, we crossed 3 founder mice (#3 carrying the Δ1–3 allele and #4 and #6 carrying the Δ2–4 allele) obtained via microinjection at the 1-cell stage. Genotyping analysis of F_1_ pups from #3 resulted in detection of the Δ1–3 allele in 50% (4 out of 8) of the pups. In addition, further genotyping of the other F_1_ generations revealed that 44% (7 out of 16) of the pups from #4 and 60% (9 out of 15) of the pups from #6 carried the Δ2–4 allele, suggesting that founder mice generated via zygotic microinjection heterozygously contributed the deleted allele (Table 2).

**Table 2:** Germline transmission of founder mice generated by the modified 2CC method.

Genotype	Injection protocol	Mouse ID	Sex	No. of genotyped F_1_ embryos	No. of Wild-type F_1_	No. of F_1_ with Deletion
Δ1–3	Standard	#3	Female	8	4	4
	2CC	#8	Male	8	6	2
		#12	Female	8	4	4
		#21	Male	7	5	2
Δ2–4	Standard	#4	Male	16	9	7
		#6	Female	15	6	9
	2CC	#3	Male	9	4	5
		#5	Female	7	7	0
		#6	Male	21	9	12
		#9	Female	9	6	3
		#11	Male	33	33	0

The same analysis was performed using F_1_ embryos produced from founder mice injected at the 2-cell stage. The results showed that 25% (2 out of 8), 50% (4 out of 8), and 29% (2 out of 7) of the embryos from #8, #12, and #21 carried the Δ1–3 allele, respectively. Similar results were also observed in founder mice carrying the Δ2–4 allele; 56% (5 out of 9), 57% (12 out of 21), and 33% (3 out of 9) of the embryos produced from #3, #6, and #9 carried the Δ2–4 allele, respectively. However, no embryos carrying the Δ2–4 alleles were obtained when founder mice #5 or #11 were used, suggesting that there were no cells with mutant alleles contributing to the germ cell pool in these founders (Table 2).

Taken together, these results demonstrate that founder mice carrying NHEJ-mediated deletion of the IG-DMR can efficiently be generated using the modified 2CC method, and these deletion alleles can be inherited by the next generation.

### Modified 2CC method can be applied to HDR-mediated knock-in

**Figure 3:**
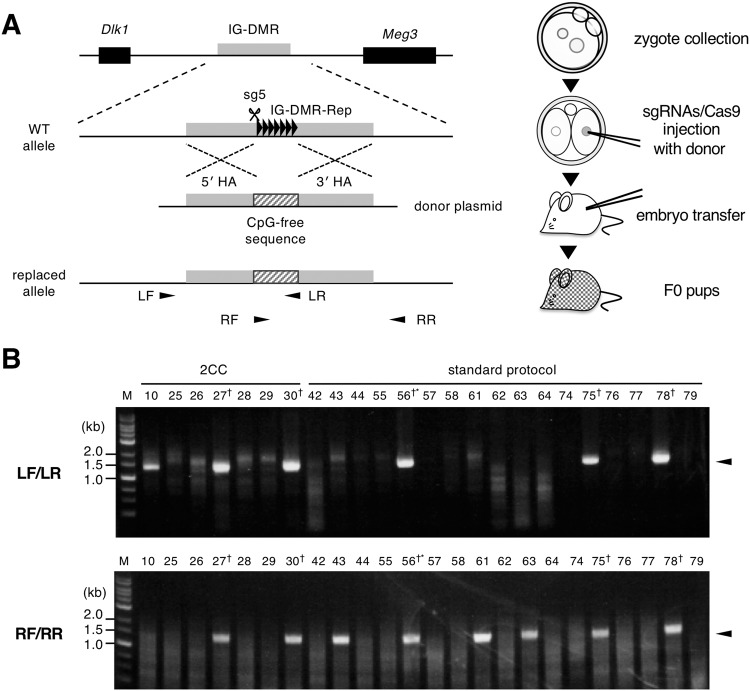
Introduction of an HDR-mediated knock-in at the IG-DMR locus using the modified 2CC method. (**A**) Schematic representation of generating knock-in allele for replacement of the IG-DMR-Rep with a CpG-free sequence. Genomic DNA, genes, and IG-DMR-Rep are indicated with a black line, boxes, and a white box, respectively. A CpG-free sequence is indicated by a dark grey box. 5′ HA and 3′ HA designate the homology arms of the donor plasmid. (**B**) Schematic representation of the experimental protocol. The sgRNA/Cas9 complex is injected into the nucleus of one blastomere at the 2-cell stage. Resulting embryos were transferred and subjected to genotyping analysis. (**C**) Genotyping analysis of the replaced allele in F_0_ pups injected with sg5/Cas9 with a targeting vector. Agarose gel electrophoresis images of PCR products amplified using LF/LR (top) and RF/RR (bottom) primer pairs are shown. Numbers indicate individual mouse IDs. ‘one blastomere injection’ and ‘1-cell’ indicate one blastomere injection at the 2-cell stage and 1-cell stage injection, respectively. A mouse that died within 10 days after birth is shown by asterisk. Daggers (†) indicate the PCR products detected by both LF/LR and RF/RR. Arrowheads indicate the estimated position of the PCR products. M: 1 kb ladder marker. Full agarose gel images for Fig. 3B are shown in Supplementary Fig. S4.

Next, we examined whether this method could be used to produce mice with HDR-mediated knock-in alleles. To test this, we attempted to generate knock-in alleles in which a tandem repeated array sequence within IG-DMR (IG-DMR-Rep) was substituted with a CpG-free sequence (IG-DMR^CG–^). As a targeting vector, a plasmid containing a 207-bp sequence of an endogenous CpG-free region and 1-kb of each homology arm flanking IG-DMR-Rep was constructed (Fig. 3A). We first injected sgRNA targeting IG-DMR (sg5) and Cas9 protein with the targeting vector into 138 zygotic pronuclei. One hundred and twenty embryos at the 2-cell stage were transferred to pseudo-pregnant females, and 46 living founder pups were obtained. From genotyping analysis of PCR products amplified using primer pairs LF/LR and RF/RR, the knock-in was identified in 3 out of 46 pups (Figs. 3B), as expected. Finally, 44 mice grew to adulthood normally, and 2 carried the IG-DMR^CG–^ allele (#75 and #78; 4.5% of the surviving pups) (Table 3).

**Table 3:** Generation of mice with knock-in at IG-DMR using the modified 2CC.

sgRNA/Cas9 combination	Injection protocol	No. of injected embryos	No. of Transferred embryos (litter no)	No. of surviving F_0_ pups (survival rate)^a^	No. of pups with knock-in^b^	No. of F_0_ pups grown to adult
sg5/Cas9 protein/donor	Standard	136	120 (4)	46 (38%)	3 (7%)	2
	2CC	93	83 (2)	40 (48%)	2 (5%)	2

^a^Survival rate indicates percentage of living pups to transferred 2-cell embryos.

^b^Parentheses indicate percentage of pups with knock-in allele to total number of surviving pups.

Next, we injected sg5/Cas9 and the targeting vector into one nucleus of 93 2-cell embryos, and then transferred embryos to pseudo-pregnant females. As a result, we obtained 40 founder pups. Genotyping analysis of these founders showed that 2 out of the 40 surviving pups (#27 and #30; 5.0% of the survived pups) carried the IG-DMR^CG–^ allele. Unlike NHEJ-mediated deletion, no significant difference in survival rate was observed between modified 2CC and the standard protocol, possibly because the NHEJ-mediated indel mutation induced by sg5/Cas9 did not cause severe lethality (Table 3). Sequencing analysis of the LF/LR amplified PCR products confirmed that all four founder mice carried the expected sequences flanking the target sg5 sequence (Supplementary Fig. S2). These results indicate that mice carrying an HDR-mediated knock-in allele can be generated using the modified 2CC method, with an efficiency similar to that observed upon direct injection of constructs into zygotes (Table 3, Figs. 3C, and S2).

To test the germline transmission of the IG-DMR^CG–^ allele, founder mice were crossed with wild-type mice. As the paternal transmission of the IG-DMR^CG–^ allele causes embryonic lethality, embryos that were transmitted paternally and newborn pups that were transmitted maternally were used for genotyping analysis at 9.5 days post coitum (dpc). As expected, genotyping analysis of F_1_ pups and embryos showed that 26% (7 out of 27) and 41% (7 out of 17) of the embryos from #75 and #78 were carrying the IG-DMR^CG–^ allele, respectively (Table 4). Similarly, 6.2% (1 out of 16) and 11% (1 out of 9) of the embryos from #27 and #30 were categorized as knock-ins, respectively (Table 4). Taken together, these results demonstrate that mice carrying an HDR-mediated knock-in alleles can be generated using the modified method.

**Table 4:** Germline transmission of knock-in mice generated using the modified 2CC.

Injection protocol	Mouse ID	Sex	No. of genotyped F_1_ pups/embryos	No. of Wild-type F_1_	No. of F_1_ with knock-in
Standard	#75	Male	27	20	7
	#78	Female	17	12	7
2CC	#27	Male	16	15	1
	#30	female	9	8	1

### IG-DMR^+/CG−^ mice exhibit loss of imprinting of the paternal IG-DMR

**Figure 4:**
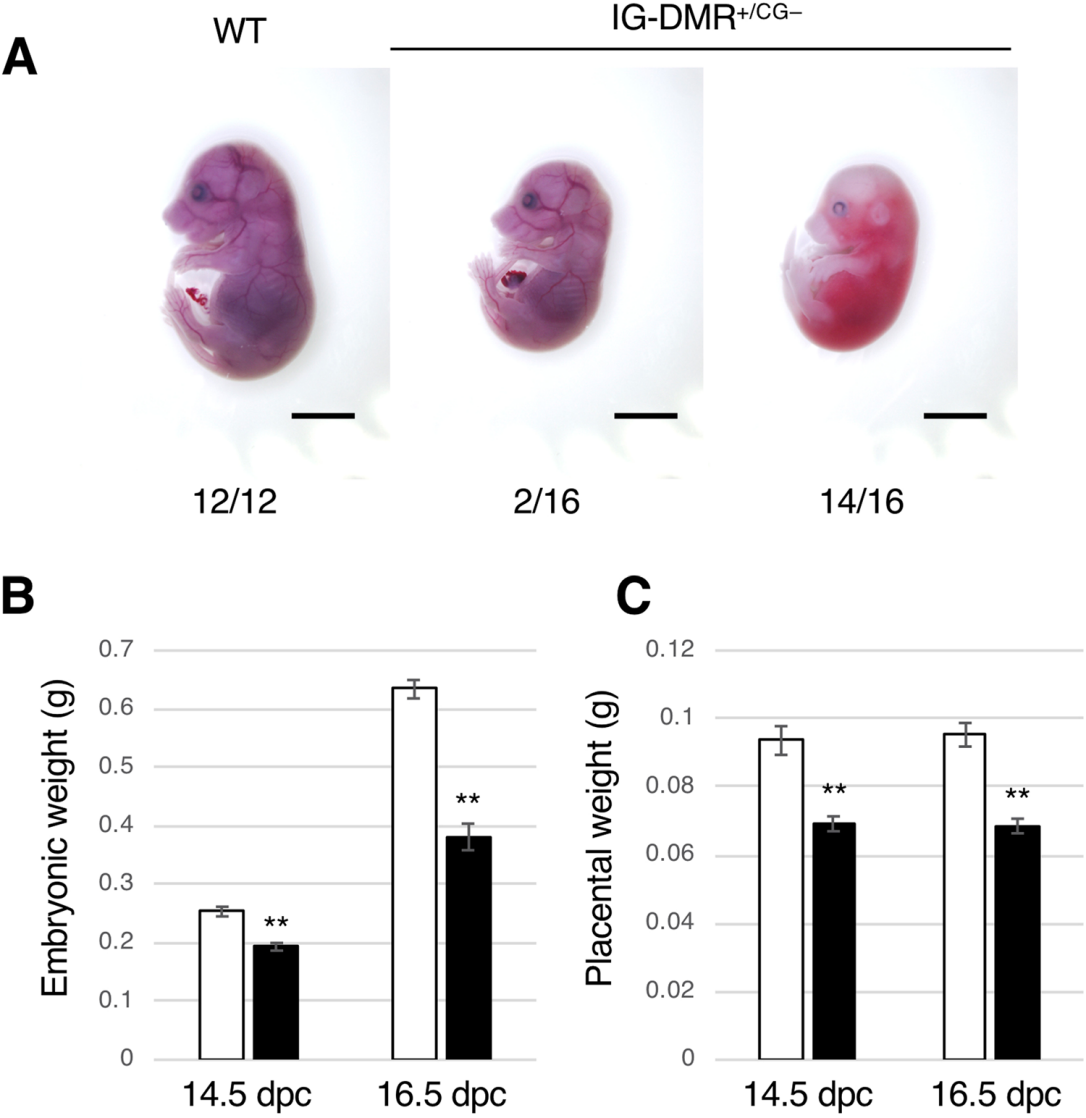
Phenotypes of the IG-DMR^+/CG−^ embryos. (**A**) Representative images of WT and IG-DMR^+/CG–^ embryos at 16.5 dpc. Numbers of embryos are indicated below the images with the corresponding embryos/total. Scale bar = 5 mm. (**B**) Body weight of WT and IG-DMR^+/CG–^ embryos at 14.5 dpc (WT and IG-DMR^+/CG–^; n = 13 and n = 15, respectively, from three litters) and 16.5 dpc (WT and IG-DMR^+/CG–^; n = 13 and n = 3, respectively, from three litters). (**C**) Placental weight (right) of WT and IG-DMR^+/CG–^ embryos at 14.5 dpc (WT and IG-DMR^+/CG−^; n = 13 and n = 15, respectively, from three litters) and 16.5 dpc (WT and IG-DMR^+/CG−^; n = 13 and n = 3, respectively, from three litters). White and black bars indicate WT and IG-DMR^+/CG–^ embryos, respectively. Error bars indicate standard error. **p < 0.01 (t-test).

To test whether the IG-DMR^CG–^ allele that we generated leads to loss of imprinting in addition to IG-DMR-Rep deletion, a knock-in allele (IG-DMR^CG–^) was transmitted paternally or maternally by crossing F_1_ mutants (derived from #30 or #78) with wild type (WT) mice. Genotyping results revealed that 11 out of 18 pups carried a maternally transmitted IG-DMR^CG–^ allele. These pups possessed body weights similar to that of WT, and they grew into adults normally, suggesting that maternal transmission of IG-DMR^CG–^ results in no obvious phenotype. In contrast, paternally transmitted IG-DMR^CG–^ alleles were not detected in any of the 14 pups obtained from three litters produced by crossing male IG-DMR^CG–^ mice with WT females. At 14.5 and 16.5 dpc, embryos possessing transmitted IG-DMR^CG–^ alleles (IG-DMR^+/CG–^) were identified in 16 out of 29 and 16 out of 28 embryos, respectively. At 14.5 dpc, 81% (13 out of 16) of IG-DMR^+/CG–^ embryos were alive; however, at 16.5 dpc, only 13% (2 out of 16) of the IG-DMR^+/CG–^ embryos were alive, with severe growth retardation, and 88% (14 out of 16) had died (Fig. 4A and Supplementary Fig. S3). Significantly decreased body and placental weights were observed in IG-DMR^+/CG–^ embryos from 14.5 dpc, suggesting that the embryonic lethality in IG-DMR^+/CG–^ embryos occurred between these stages (Fig. 4B).

**Figure 5:**
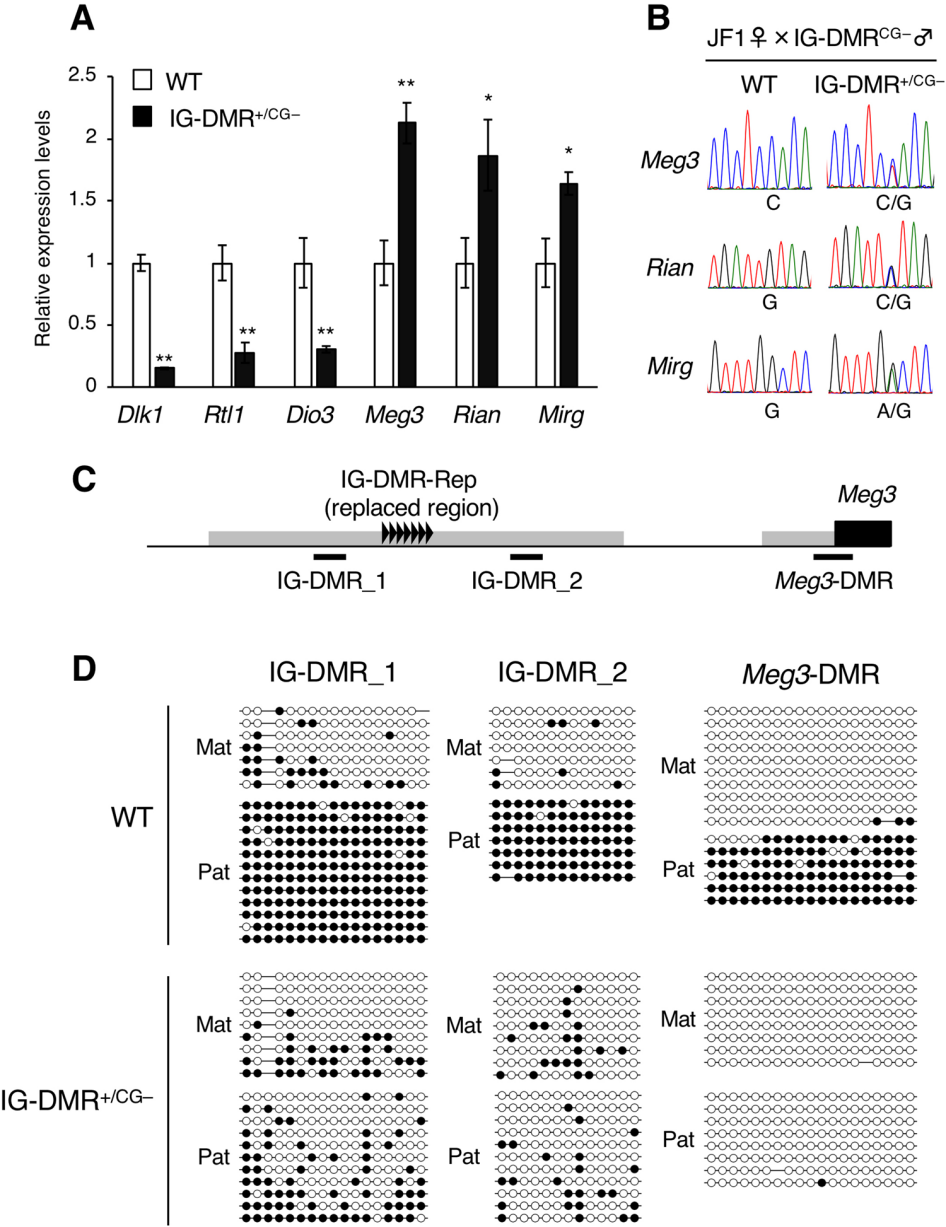
Imprinting status of the *Dlk1*-*Dio3* domain in IG-DMR^+/CG–^ embryos. (**A**) Quantitative RT-PCR analysis of WT and IG-DMR^+/CG–^ embryos at 14.5 dpc (WT and IG-DMR^+/CG–^; n = 8 and n = 7, respectively, from two litters). White and black bars indicate WT and IG-DMR^+/CG–^ embryos, respectively. Error bars indicate standard error. **p < 0.01 (t-test). (**B**) Allelic expression of the maternally imprinted genes in WT and IG-DMR^+/CG–^ embryos at 14.5 dpc. A representative electropherogram of the RT-PCR products is shown. (**C**) Schematic representation of the PCR amplified regions used for DNA methylation analysis. Genomic DNA is indicated with a black line. IG-DMR and *Meg3*-DMR are indicated with gray boxes. IG-DMR-Rep (replaced region) is indicated with black triangles. *Meg3* gene is indicated by a black box. IG-DMR_1, IG-DMR_2 and *Meg3*-DMR are shown with bold black lines. (**D**) DNA methylation status of IG-DMR_1, IG-DMR_2 and *Meg3*-DMR. Representative methylation status of WT and IG-DMR^+/CG–^ embryos at 14.5 dpc is presented. Opened and closed circles indicate unmethylated and methylated CpG, respectively.

Expression analysis of imprinted genes in IG-DMR^+/CG–^ embryos revealed the repression of paternally expressed genes (*Dlk1*, *Rtl1*, and *Dio3*) and the overexpression of maternally expressed genes (*Meg3*, *Rian*, and *Mirg*) (Fig. 5A). Allelic expression analyses of *Meg3*, *Rian*, and *Mirg* using inter-subspecies polymorphisms between C57BL/6 and JF1/Ms showed that these genes were biallelically expressed in IG-DMR^+/CG–^ embryos (Fig. 5B). Similarly, DNA methylation analysis of two IG-DMR regions (IG-DMR_1 and IG-DMR_2) and the promoter CpG of the *Meg3* gene (*Meg3*-DMR, Fig. 5C) revealed that all regions on the paternal allele were fully hypomethylated, suggesting that aberrant expression of imprinted genes occurred due to the loss of paternal methylation imprints in the IG-DMR and *Meg3*-DMR (IG-DMR_1: WT vs IG-DMR^+/CG–^; 96% vs 37%, respectively; P < 0.01, IG-DMR_2: WT vs IG-DMR^+/CG–^; 98% vs 17%, respectively; P < 0.01, Meg3-DMR: WT vs IG-DMR^+/CG–^; 89% vs 1%, respectively; P < 0.01) (Fig. 5D).

Taken together, these results indicate that the phenotype of mice with allelic replacements that were generated using the modified 2CC method recapitulates that of IG-DMR-Rep deletion.

## Discussion

In the present study, we demonstrated that NHEJ-mediated deletion and knock-in mutations in loci that are essential for normal development can be generated by genome editing of the blastomere of 2-cell embryos, possibly through mosaicism of mutated and wild-type cells. In a previous study, we obtained only a single mouse carrying a deleted IG-DMR-Rep from more than 2,000 TALEN-injected embryos. Additionally, we failed to establish a deletion line from this single male founder, as almost all embryos paternally transmitted the deletion allele and were embryonic lethal^11^. Therefore, an efficient method to produce heritable founder mice carrying a modified allele is required to effectively study the regulatory mechanisms of the IG-DMR. Our results showed that a modified 2CC method is useful for generating mice genetically modified at the IG-DMR locus with higher survival rates than the standard protocol.

Also, we showed that modified alleles in the founder mice can be efficiently transmitted to the next generation. Founder mice generated using the standard protocol transmitted deletion and WT alleles at a 1:1 ratio, suggesting that these mice were heterozygous mutants for the IG-DMR deletion. Further, mutant mice generated using the modified 2CC method, such as #12 (for Δ1–3) and #3 (for Δ2–4), also transmitted deletion and WT alleles at a 1:1 ratio but were considered mosaic mice, carrying both cells with the homozygous deletion and WT cells. Since homozygous Δ1–3 deletion is embryonic lethal at late-stage gestation, it is reasonable to conclude that the lethality of homozygous deletion of IG-DMR was rescued by wild-type cells in the mosaic mice. However, we also showed that the contribution of cells with mutated alleles to the germline of mice generated using the modified 2CC method was different from one individual to the next. Some founder mice exhibited different genotypes between analyses using a fingertip and a tail tip. Also, mutated alleles identified in a fingertip could not be transmitted to the F_1_ generation. To obtain F_1_ mice carrying modified alleles, it was safest to use several founder mice for mating with wild-type mice.

The 2CC method is an efficient method for generating mutant mice that carry lethal mutations by forcibly inducing mosaicisms that help wild-type cells rescue individuals from the lethal effects of mutated cells. However, several problems can arise if the heterozygous mutation causes cell-autonomous apoptosis, ceases cell division, causes loss of stemness, or halts germ cell differentiation, among others. One example is a mutation in the Y-linked gene *Eif2s3y*. Knock out of the gene causes male infertility due to the meiotic arrest of germ cells and induction of apoptosis^18^. In this case, we would assume that a mosaic mutant consisting of *Eif2s3y*-KO/wild-type generated using the 2CC method would produce sperm derived from wild-type cells only, and not from mutant cells, because germ cells carrying the mutation would still undergo meiotic arrest. In addition, there is a possibility that toxicity of the injected sgRNA, Cas9, or donor template DNA would affect the contribution of mutated cells in mosaic founder mice. We note that, at least in this study, microinjection of sgRNA/Cas9 did not affect the contribution of mutated cells, as the embryos injected with sg1/sg3/Cas9 protein at the 1-cell stage were obtained at 16.5 dpc.

Previously, we reported that deletion of the IG-DMR-Rep caused a loss of imprinting on the paternal allele of IG-DMR^11^; however, we could not distinguish whether the loss of imprinting resulted from a loss of methylated IG-DMR-Rep or from a conformational change in the IG-DMR. Our results indicated that the phenotype of IG-DMR^+/CG–^ was the same as that observed in the IG-DMR-Rep deleted embryos, implying that the loss of imprinting in the IG-DMR-Rep deletion embryos was caused by a loss of methylated IG-DMR-Rep. This result indicates differences in ICR function between IG-DMR and *H19*-ICR. Szabó *et al*. reported that the replacement of *H19*-ICR with an exogenous sequence caused loss of paternal methylation imprints in *H19*-ICR. Methylation imprinting of the exogenous sequence was not established during male germ cell development, suggesting that the 2.4-kb substituted region of *H19*-ICR contains a region essential for establishing DNA methylation imprint^19^. Our previous study showed that IG-DMR was completely methylated in germ cells of mice with deleted IG-DMR-Rep, suggesting that IG-DMR-Rep is essential for the maintenance of methylation imprints but not establishment^11^. In both cases, specific sequences are required to maintain the imprinting status of ICRs throughout embryonic development.

In summary, the modified 2CC method for generating artificial mosaicism is useful for producing mutant mice with alleles that cause embryonic lethality, using the CRISPR/Cas9 system via both NHEJ-mediated deletion and HDR-mediated knock-in. In addition, the modified 2CC method may contribute to reductions in number of recipient zygotes and foster mothers for obtaining founder pups.

## Materials and Methods

### sgRNA target sequences

Recognition sequences of the sgRNAs used in this study are as follows: sg1: 5′-ACACACGGTCCGTTACAGCCTGG-3′; sg2: 5′-GTCGATCGTGAACTGCAGCCTGG-3′; sg3: 5′-GGAGAATGCCTTGAGCACAGGGG-3′; sg4: 5′-AGGAGAAACCACTATAGCGTTGG-3′; and sg5: 5′-TTCGCTATGAACTACCGCTACGG-3′. PAM sequences are underlined.

### Preparation of sgRNA, Cas9, and targeting vector

sgRNA cloning vector and human codon-optimized Cas9 (hCas9) plasmids were.pngted by George Church (Addgene plasmid #41824 and #41815, respectively)^2^. sgRNAs were cloned into a sgRNA cloning vector as described in previous reports^20^. sgRNAs and Cas9 were transcribed *in vitro* using the mMESSAGE/mMACHINE T7 Transcription Kit (Ambion, Austin, TX, USA). RNAs were purified using the MEGAclear Transcription Clean-Up Kit (Ambion). Primers listed in the Supplementary Table were used for cloning of sgRNAs and template amplification in *in vitro* transcription reactions.

To construct the targeting vector, an endogenous CpG-free sequence (Chr8:43,288,882-43,289,076, GRCm38/mm10) was amplified from the C57BL/6 genome and cloned into the BamHI/HindIII sites of a pBluescript II KS(–) vector. Subsequently, 5′ and 3′ homology arms were amplified from the C57BL/6 genome and cloned into XbaI/BamHI sites and HindIII/SalI sites in the vector, respectively. The primers used for amplification of each sequence are listed in the Supplementary Table.

### Microinjection

For microinjection, fertilized eggs were collected from super-ovulated F_1_ hybrids of C57BL/6 and DBA/2 (BDF1) female mice crossed with BDF1 male mice. Embryos were incubated in KSOM medium (ARK resource, Kumamoto, Japan) at 37 °C overnight. For NHEJ-mediated deletion, a mixture of two sgRNAs (250 ng/µl each) and the Cas9 protein (Nippon Gene, Tokyo, Japan; 100 ng/µl) was microinjected into the cytoplasm of one blastomere of the 2-cell embryo. As a control, sgRNAs and Cas9 mRNA (167 ng/µl each) were injected into the cytoplasm of fertilized eggs as previously described^21^.

For HDR-mediated knock-in, a mixture of the sgRNA (250 ng/µl), Cas9 protein (100 ng/µl), and the targeting vector (5 ng/µl) was similarly injected. Injected 2-cell embryos were incubated in KSOM medium at 37 °C for 1 hour, then transferred into pseudo-pregnant ICR female mice. All mice were purchased from Sankyo Lab Service (Tokyo, Japan). All animal protocols were approved by the Animal Care and Use Committee of the National Research Institute for Child Health and Development, Tokyo, Japan. All experiments were conducted in accordance with the approved animal protocols.

### Genotyping analysis

To genotype the founder pups and embryos, genomic DNA was extracted from the fingertips or tail tips of founders. PCR was performed by BIOTAQ (BioLine, London, UK), using the primers listed in the Supplementary Table. For sequencing analysis, PCR products were treated with ExoSAP-IT (Affimetrix, Santa Clara, CA, USA) and sequenced on a capillary sequencer (3130xl and 3500xL Genetic Analyzers, Applied Biosystems, Foster City, CA, USA).

### Expression analysis

Expression analyses were performed as previously described^10^. Briefly, total RNA was isolated from embryos at 14.5 dpc using ISOGEN (Nippon Gene), then treated with TURBO DNase (Ambion). cDNA was synthesized by Superscript II (Thermo Scientific) using a random primer (for Rtl1) or an oligo dT primer (for other genes). Quantitative PCR was performed using Power SYBR Green (Thermo Scientific) and primers as previously described.

To detect the allelic expression of imprinted genes, IG-DMR^+/CG–^ embryos at 14.5 dpc were obtained by crossing IG-DMR^CG–^ male and wild-type JF1/Ms female mice. RT-PCR products amplified with ExTaq HS (Takara Bio, Shiga, Japan) were treated with ExoSAP-IT and sequenced. JF1/Ms mice were obtained from the National Institute of Genetics.

### DNA methylation analysis

Genomic DNA was extracted from the tail tip of embryos at 14.5 dpc. Sodium bisulfite conversion of DNA was performed using the EZ DNA methylation kit (Zymo Research, Irvine, CA, USA). PCR was performed using primers targeting IG-DMR_1, IG-DMR_2, and *Meg3*-DMR and EpiTaq HS (Takara Bio). Amplified PCR products were cloned into a pGEM-T easy vector (Promega) and sequenced. Sequence data was analyzed by QUMA (http://quma.cdb.riken.jp/top/quma_main_j.html)^22^.

### Statistical analyses

All experiments were performed using at least two biological replicates. Significant differences between survival rates of the embryos were determined by Fisher’s exact test. For analysis of the embryo/placental weights and mRNA expression levels, Student’s *t*-test was used. DNA methylation levels were analyzed using Mann-Whitney’s U-test (included in QUMA). *P*-values less than 0.05 were considered significant.

## Supplementary information

Supplementary Information
