# Supplementary material for: Efficient production and transmission of CRISPR/Cas9-mediated mutant alleles at the IG-DMR via generation of mosaic mice using a modified 2CC method
Source: Sci Rep. 2019 Dec 27;9:20202. doi: 10.1038/s41598-019-56676-5 (PMC6934616; doi:10.1038/s41598-019-56676-5)
Supplement: Supplementary file 1 — Supplementary Information [file 41598_2019_56676_MOESM1_ESM.pdf]

Efficient production and transmission of CRISPR/Cas9-mediated mutant alleles at the IG-DMR via generation of mosaic mice using a modified 2CC method

Satoshi Hara[1,2,*], Miho Terao[1], Akari Muramatsu[1] and Shuji Takada[1,*]

[1]Department of Systems BioMedicine, National Research Institute for Child Health and Development, Tokyo 157-8535, Japan
[2]Division of Molecular Genetics & Epigenetics, Department of Biomolecular Sciences, Faculty of Medicine, Saga University, Saga 849-8501, Japan

*Correspondence should be addressed to Satoshi Hara (shara@cc.saga-u.ac.jp, 5-1-1 Nabeshima, Saga 849-8501 Japan, Tel: +81-952-34-2264) and Shuji Takada (takada-s@ncchd.go.jp, 2-10-1 Okura, Setagaya, Tokyo 157-8535, Japan, Tel & Fax: +81-3-3417-2498)

Figure S1

```
F1/R3
                            sg1                                        sg3
WT      CAGCACATACACACGGTCCGTTACAGCCTGG.............TTTTGTCAATGGAGAATGCCTTGAGCACAGGGG
#8      CAGCACATACACACGGTCCA-------------(1820 bp)----------ATGGAGAATGCCTTGAGCACAGGGG
#12     CAGCACATACACACGGTCCGTTACA--------(1813 bp)--------CAATGGAGAATGCCTTGAGCACAGGGG
#12     CAGCACATACACACGGTCCGTTACA--------(1815 bp)----------ATGGAGAATGCCTTGAGCACAGGGG
#21     CAGCACATACACACGGTCCGTTACA--------(1813 bp)--------CAATGGAGAATGCCTTGAGCACAGGGG
#22     CAGCACATACACACGGTCCGTTACA--------(1813 bp)--------CAATGGAGAATGCCTTGAGCACAGGGG
#24     CAGCACATACACACGGTCCGTTACA--------(1812 bp)-------TCAATGGAGAATGCCTTGAGCACAGGGG
```

```
F2/R4
                                             sg2                                      sg4
WT      GTGAATCTATACGGAGATGTGCTGTGGACCCCAGGCTGCAGTTCACGATCGAC.............AGCCCAGGAGAAACCACTATAGCGTTGGTTGCCGT
#2      GTGAATC----------------------------------------------..(3077 bp)..-----------------------GTTGGTTGCCGT
#5      GTG--------------------------------------------------..(3085 bp)..---------------------------GTTGCCGT
#6      GTGAATacggagatgtgctgtggaccctcaaaaagccag--------------..(3045 bp)..-----------------------GTTGGTTGCCGT
#9      GTGAATCT---------------------------------------------..(3075 bp)..----------------------CGTTGGTTGCCGT
#12     GTGAATCT---------------------------------------------..(3075 bp)..----------------------CGTTGGTTGCCGT
```

Figure S1. Nucleotide sequences of deleted alleles in pups generated using the modified 2CC method. Sequences of PCR products amplified with primer pairs F1/R3 and F2/R4 are shown. Wild-type (WT) sequence is indicated at the top as a reference. Mouse IDs are shown at left. Each target sequence of sgRNA is underlined. Deleted nucleotides are shown with hyphens. Unexpected insertion is indicated with small letters. Parentheses show number of deleted nucleotides.

Figure S2

5′ HA IG-DMR-Rep

WT 5′…TACTGTACACAATGCTGCCGTTCGCTATGAACTACCGCTACGGTTCATAGT…3′

IG-DMR^{CG-} 5′…TACTGTACACAATGCTGCggatccTGTGTAGGAGAAGATGACAGAAGCTAG…3′

5′ HA BamHI CpG-free sequence

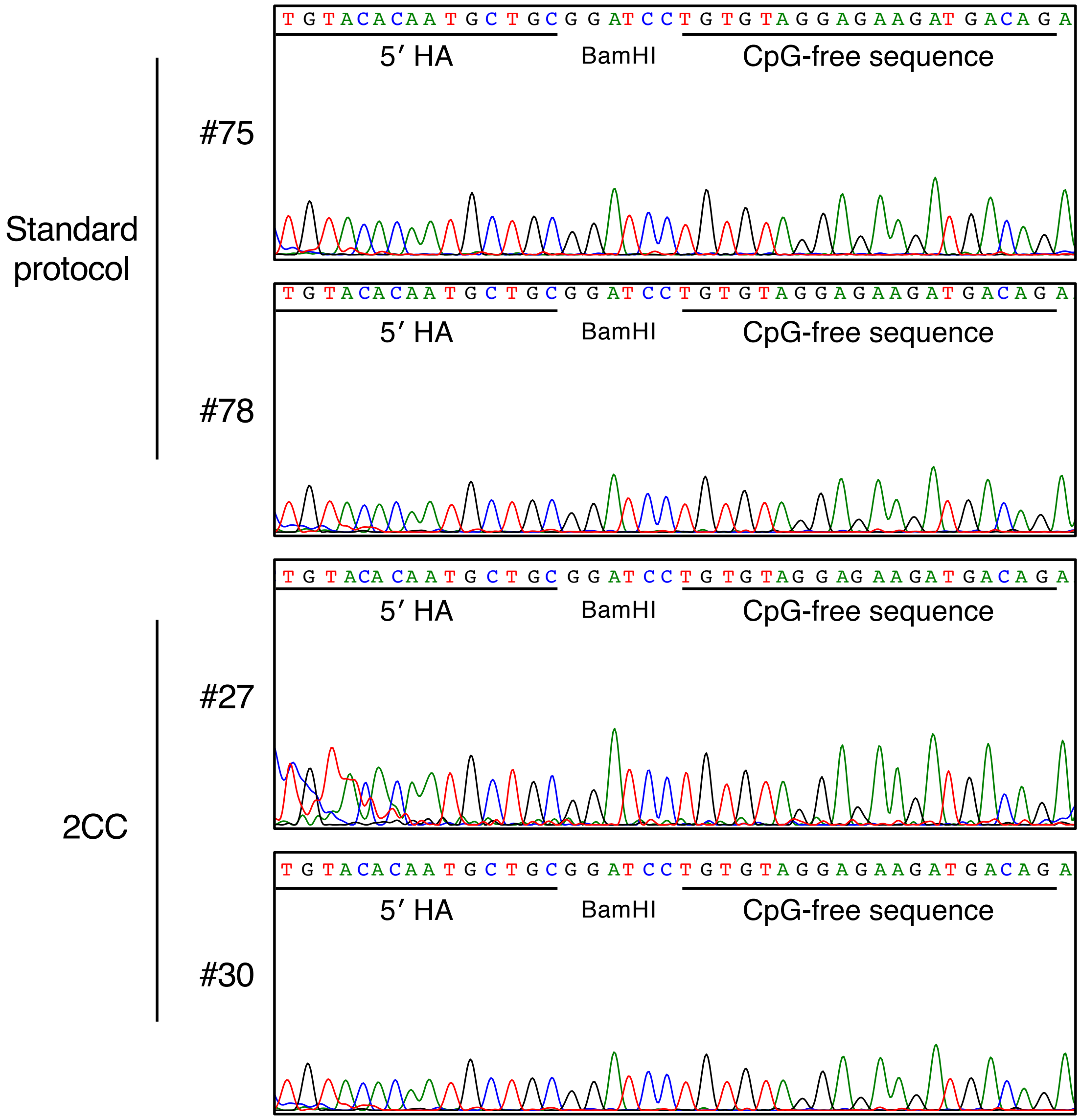

Figure S2. Nucleotide sequences of the IG-DMR^{CG-} alleles in founder mice generated using the modified 2CC method.
(top) Nucleotide sequences surrounding sg5. Wild-type (WT) and replaced (IG-DMR^{CG-}) alleles are shown. The sequence of the IG-DMR-Rep and the CpG-free sequence are highlighted in black and grey, respectively. The target sequence of sg5 is underlined. The BamHI recognition sequence is shown using lowercase letters. 5′ HA designates a homology arm sequence within the donor DNA template. (bottom) Electropherograms of founder mice generated by the standard protocol and modified 2CC method. Electropherograms were obtained by direct sequencing of PCR products amplified using the LF/LR primers.

Figure S3

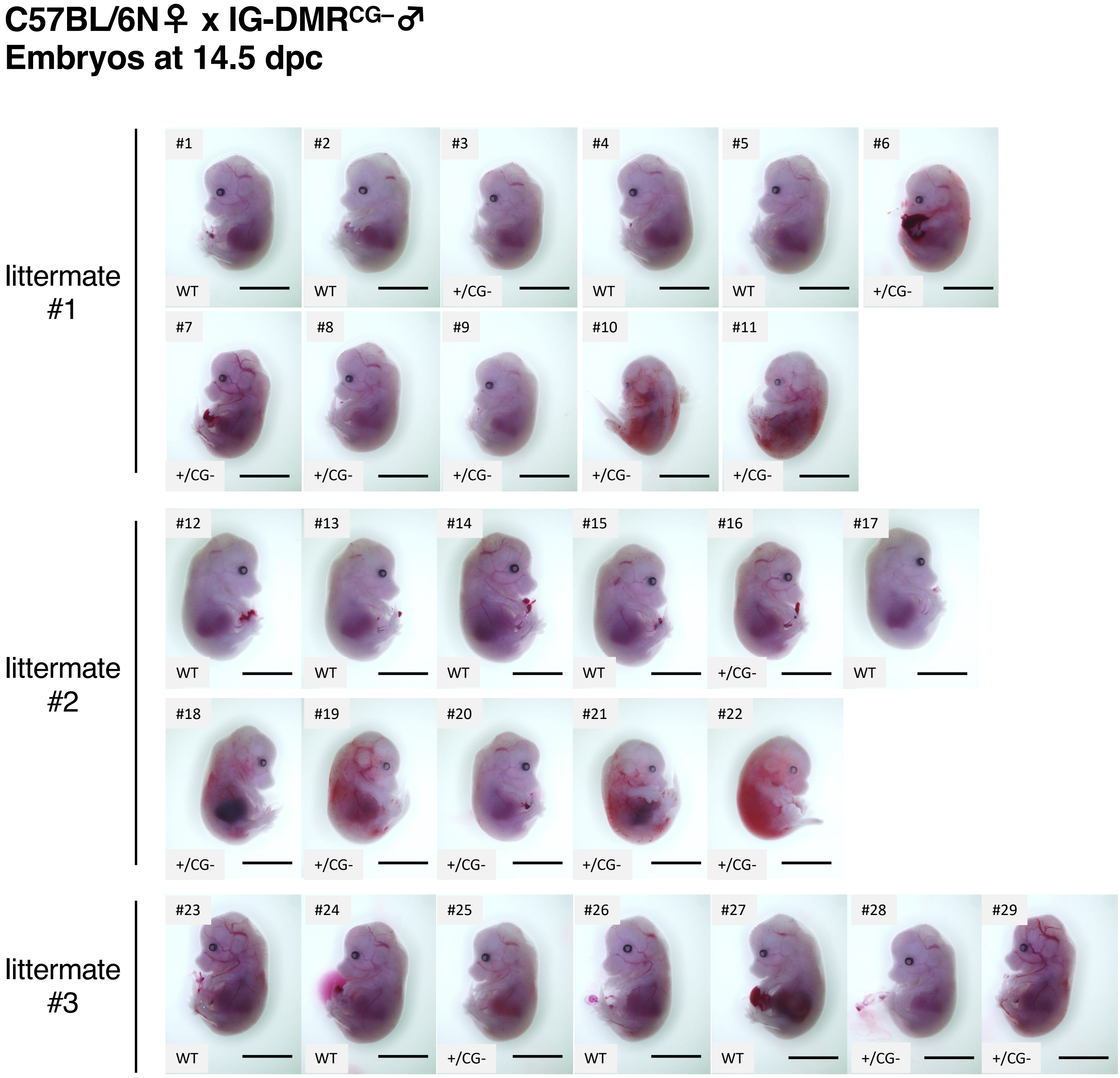
C57BL/6N♀ x IG-DMRCG−♂
Embryos at 14.5 dpc
littermate #1
#1 WT
#2 WT
#3 +/CG-
#4 WT
#5 WT
#6 +/CG-
#7 +/CG-
#8 +/CG-
#9 +/CG-
#10 +/CG-
#11 +/CG-
littermate #2
#12 WT
#13 WT
#14 WT
#15 WT
#16 +/CG-
#17 WT
#18 +/CG-
#19 +/CG-
#20 +/CG-
#21 +/CG-
#22 +/CG-
littermate #3
#23 WT
#24 WT
#25 +/CG-
#26 WT
#27 WT
#28 +/CG-
#29 +/CG-

Figure S3 (continued)

C57BL/6N♀ x IG-DMR^{CG-}♂
Embryos at 16.5 dpc

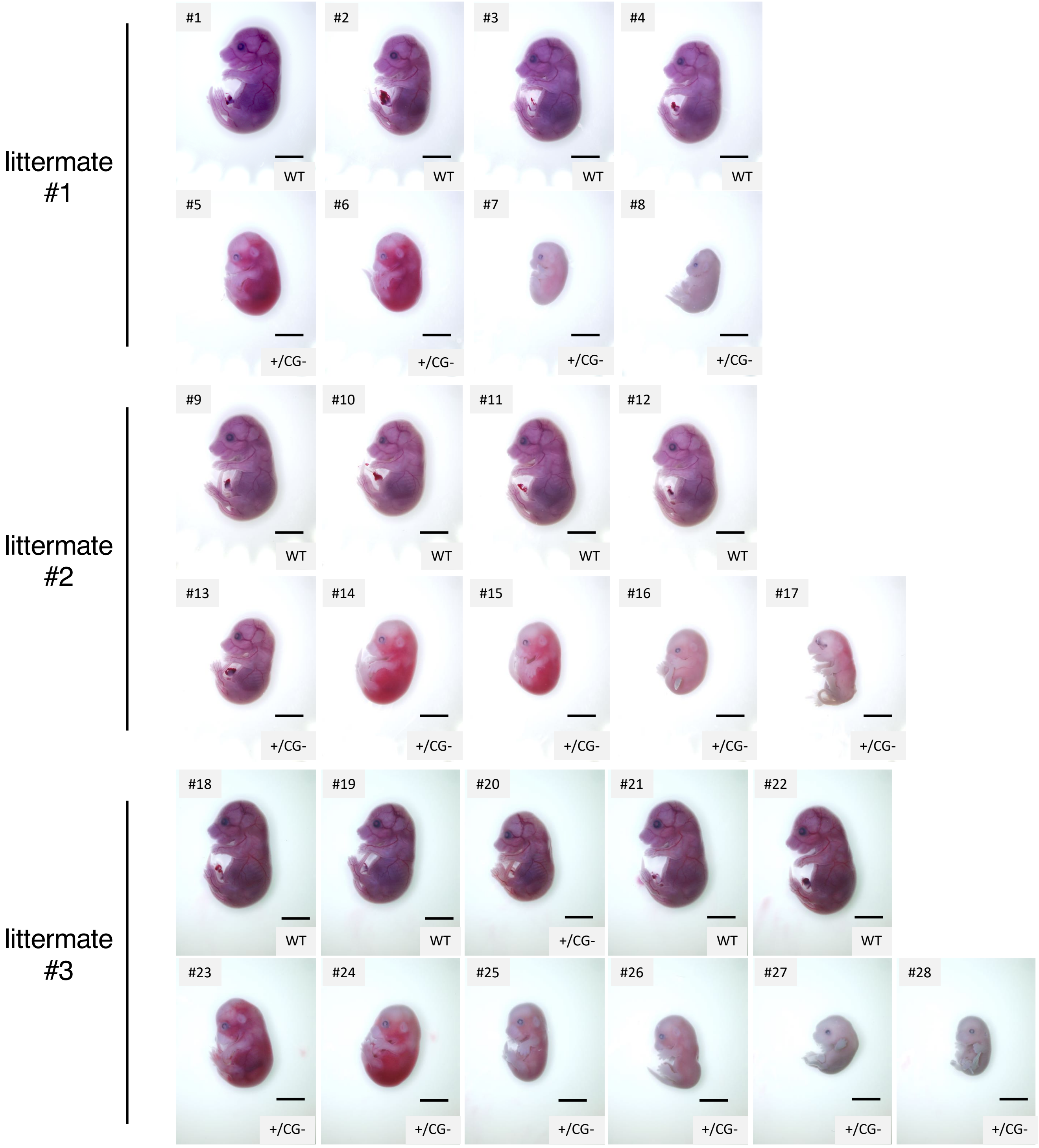

Figure S3. Embryos obtained from C57BL/6N female mice crossed with IG-DMR^{CG-} males. Photographs of embryos at 14.5 and 16.5 dpc from three independent litters. ID and genotype of individual embryos are shown in gray boxes.

Figure S4

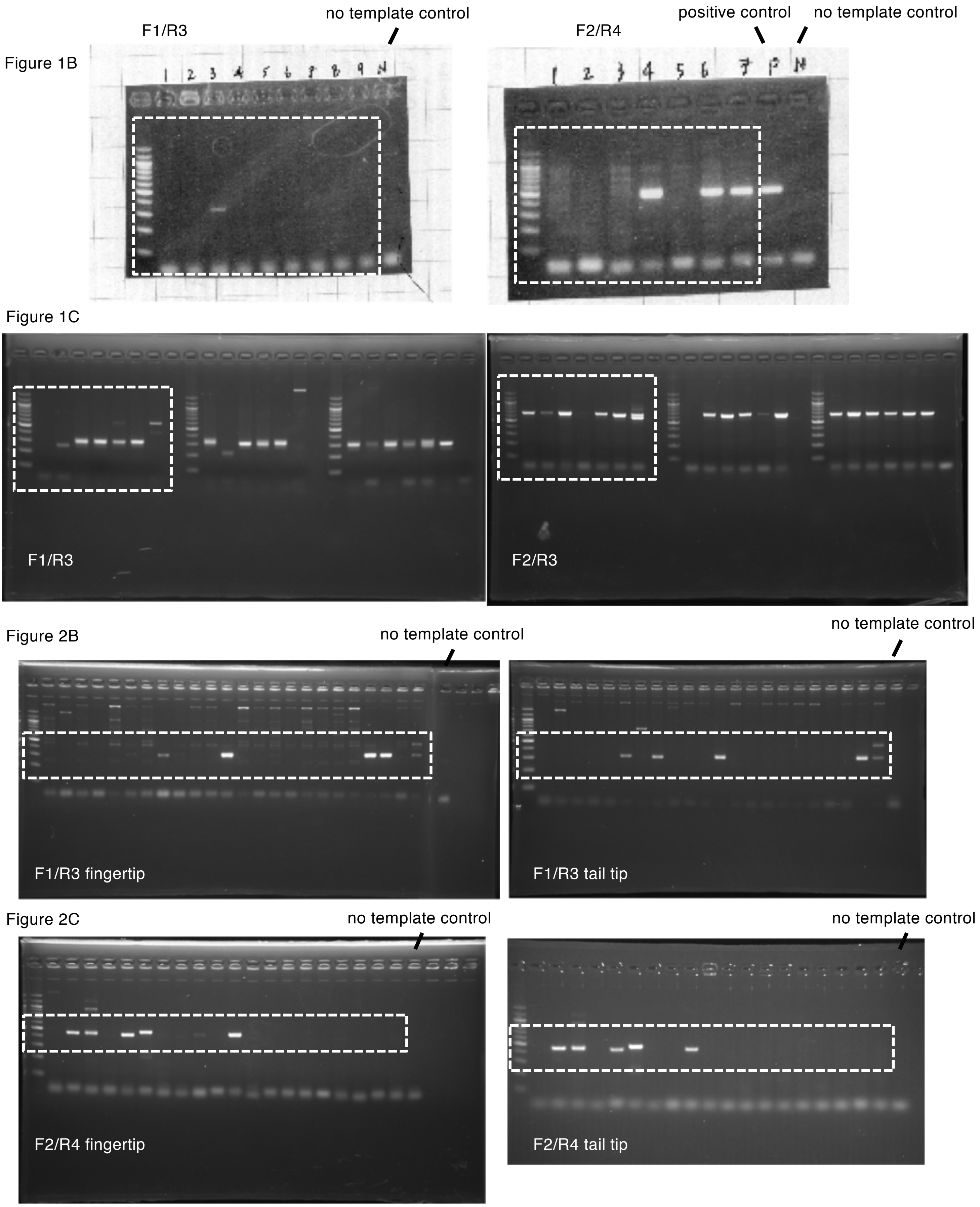
Figure 1B
F1/R3
no template control
F2/R4
positive control
no template control
Figure 1C
F1/R3
F2/R3
Figure 2B
no template control
F1/R3 fingertip
no template control
F1/R3 tail tip
Figure 2C
no template control
F2/R4 fingertip
no template control
F2/R4 tail tip

Figure S4 (continued)

Figure 3C

LF/LR

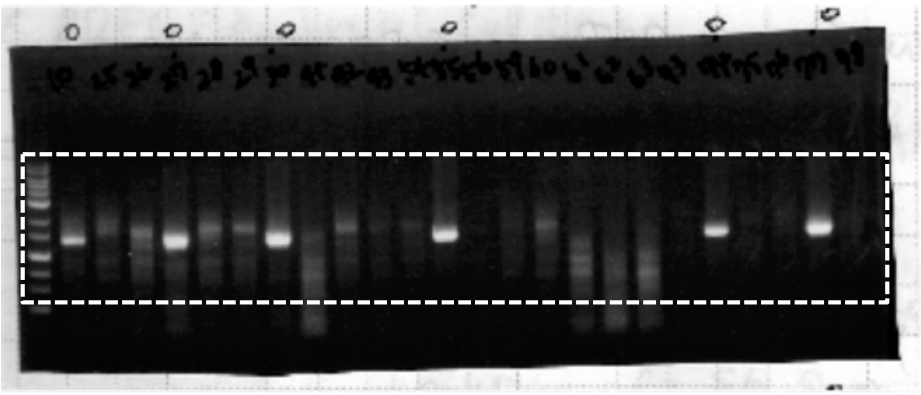

RF/RR

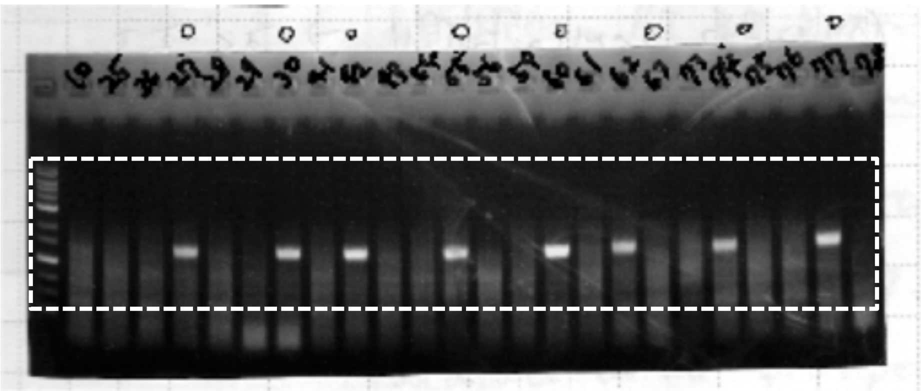

Figure S4. Original full agarose gel images shown in other figures.
Cropped regions are indicated with dashed white boxes.

Supplementary Table. Primer sequences

Procedure	Primer name	Primer sequence (5′-3′)
sg1	sg1_F	CACACGGTCCGTTACAGCCgttttagagctagaaatagcaag
	sg1_R	aacGGCTGTAACGGACCGTGTGcggtgtttcgtcctttccac
	T7_sg1	ttaatacgactcactataggCACACGGTCCGTTACAGCC
sg2	sg2_F	TCGATCGTGAACTGCAGCCgttttagagctagaaatagcaag
	sg2_R	aacGGCTGCAGTTCACGATCGAcggtgtttcgtcctttccac
	T7_sg2	ttaatacgactcactataggTCGATCGTGAACTGCAGCC
sg3	sg3_F	GAGAATGCCTTGAGCACAGGTTTTAGAGCTAGAAATAGCAAG
	sg3_R	AACCTGTGCTCAAGGCATTCTCCGGTGTTTCGTCCTTTCCAC
	T7_sg3	TTAATACGACTCACTATAGGGAGAATGCCTTGAGCACAG
sg4	sg4_F	GGAGAAACCACTATAGCGTgttttagagctagaaatagcaag
	sg4_R	aacACGCTATAGTGGTTTCTCCcggtgtttcgtcctttccac
	T7_sg4	ttaatacgactcactataggGGAGAAACCACTATAGCGT
sg5	sg5_F	TCGCTATGAACTACCGCTAgttttagagctagaaatagcaag
	sg5_R	aacTAGCGGTAGTTCATAGCGAcggtgtttcgtcctttccac
	T7_sg5	ttaatacgactcactataggTCGCTATGAACTACCGCTA
Cas9	T7-Cas9-F	TAATACGACTCACTATAGGGAGAATGGACAAGAAGTACTCCATTGG
	Cas9-R	TCACACCTTCCTCTTCTTC
5′HA	Xba_5arm_F	GCtctagaCAAATGCCTGGCATCACAGATATAT
	BamHI_5arm_R	GCggatccGCAGCATTGTGTACAGTAAGCCATG
3′HA	HindIII_3arm_F	GCaagcttAGCGCTGCAGCCGCTATGCTATGCT
	SalI_3arm_R	GCgtcgacGCAGACACCATCCCACGAGGAACTG
CpG-free sequence	BamHI_intergenic_F	GCggatccTGTGTAGGAGAAGATGACAGAAGCT
	HindIII_intergenic_R	GCaagcttAAATTTAAACATCTGGAAAAGCCAT
Genotyping	IG-DMR_1F	AAGTAACAGGCTCTCACTGG
	IG-DMR_2F	GAGTCCTATCATCCTGTATG
	IG-DMR_1R	GTCTGTATGGTCACAGCACC
	IG-DMR_2R	GCTCTGGGTGATCCACCATA
	IG-DMR_KI_LF	TGACACAGGCATAGCAAACC
	IG-DMR_KI_LR	AAATTTAAACATCTGGAAAAGCCAT
	IG-DMR_KI_RF	TGTGTAGGAGAAGATGACAGAAGCT
	IG-DMR_KI_RR	CAATCCCACCACAGCTTCTT
Methylation analysis	IG-DMR_R1_BSF	GGTTTGGTATATATGGATGTATTGTAATATAGG
	IG-DMR_R1_BSR	ATAAAACACCAAATCTATACCAAAATATACC
	IG-DMR_R2_BSF	GGATGGTAGTAGATAATTTGTTGTTTGA
	IG-DMR_R2_BSR	CCCCCAATAACTTATAAACCATAATACT
